# FasL is a catabolic factor in alveolar bone homeostasis

**DOI:** 10.1111/jcpe.13750

**Published:** 2022-11-25

**Authors:** Karol Alí Apaza Alccayhuaman, Patrick Heimel, Jung Seok Lee, Stefan Tangl, Ulrike Kuchler, Julie Marchesan, Layla Panahipour, Stefan Lettner, Eva Matalová, Reinhard Gruber

**Affiliations:** ^1^ Department of Oral Biology University Clinic of Dentistry, Medical University of Vienna Vienna Austria; ^2^ Karl Donath Laboratory for Hard Tissue and Biomaterial Research University Clinic of Dentistry, Medical University of Vienna Vienna Austria; ^3^ Department for Bioimaging Ludwig Boltzmann Institute for Traumatology, The Research Center in Cooperation With AUVA Vienna Austria; ^4^ Austrian Cluster for Tissue Regeneration Vienna Austria; ^5^ Department of Periodontology Research Institute for Periodontal Regeneration, College of Dentistry, Yonsei University Seoul Republic of Korea; ^6^ Department of Oral Surgery University Clinic of Dentistry, Medical University of Vienna Vienna Austria; ^7^ Division of Comprehensive Oral Health Adams School of Dentistry, University of North Carolina at Chapel Hill Chapel Hill North Carolina USA; ^8^ Laboratory of Odontogenesis and Osteogenesis Institute of Animal Physiology and Genetics, Czech Academy of Sciences Brno Czech Republic; ^9^ Department of Periodontology School of Dental Medicine, University of Bern Bern Switzerland

**Keywords:** FasL, gld, ligature, osteolysis, periodontitis

## Abstract

**Aim:**

Fas ligand (FasL) belongs to the tumour necrosis factor superfamily regulating bone turnover, inflammation, and apoptosis. The appendicular and axial skeleton phenotype of mature Fasl^*gld*^ mice has been reported. The impact of FasL on the alveolar bone providing support for the teeth at mature stages under healthy and induced inflammatory conditions remains unknown.

**Materials and Methods:**

We performed a phenotypical analysis of mice carrying the homozygous Fasl^*gld*^ mutation and wild‐type (WT) mice (C57BL/6) under healthy conditions and upon ligature‐induced periodontitis. After 12 days, micro‐computed tomography analysis revealed the distance between the cement enamel junction and the alveolar bone crest. Additional structural parameters, such as the bone volume fraction (BV/TV) and the periodontal ligament space volume, were measured. Histological analyses were performed to visualize the catabolic changes at the defect site.

**Results:**

Healthy Fasl^*gld*^ mice were found to have more periodontal bone than their WT littermates. Fasl^*gld*^ had no significant effect on inflammatory osteolysis compared to WT controls with ligatures. Histology revealed eroded surfaces at the root and in the inter‐proximal bone in both strains.

**Conclusions:**

Our findings suggest that FasL is a catabolic factor in alveolar bone homeostasis but it does not affect the inflammatory osteolysis.

**Clinical Relevance**

*Scientific rationale for study*: Periodontal disease challenges the scientific community to seek a molecular strategy that aims to reduce inflammatory osteolysis. Targeting Fas ligand (FasL) might be a promising therapeutic approach.

*Principal findings*: FasL plays a catabolic role in alveolar bone homeostasis in our mouse model. There was, however, no statistical effect of FasL under inflammatory conditions.

*Practical implications*: Targeting FasL may support periodontal bone homeostasis but is not a key target to protect the bone from inflammatory osteolysis.

## 1 INTRODUCTION

Fas ligand (FasL) (CD178; CD95L; APO1L), a transmembrane protein member of the tumour necrosis factor (TNF) family which interacts with the Fas (CD95; APO‐1; TNFRSF6) receptor, plays an important role in the regulation of cell death (Suda et al., 1993). The FasL/Fas pathway ignites the extrinsic apoptotic machinery of immune cells (Griffith et al., 1995; Tsutsui et al., 1996; Nagata, 1997) but may extend towards affecting bone. There is evidence for a catabolic function, as mice carrying the Fasl^*gld*^ mutation, causing a generalized lymphoproliferative disorder (Ramsdell et al., 1994), have an increased femoral bone mass compared to their wild‐type (WT) littermates (Katavic, Lukic, et al., 2003). Further support for a catabolic role of FasL is based on the alveolar bone of 24‐day‐old Fasl^*gld*^ mice having increased bone volume and trabecular thickness (Svandova et al., 2019). Thus, the Fasl^*gld*^ mutation causes a positive remodelling shift culminating in increased bone mass. Contrasting findings showed that Fasl^*gld*^ had reduced bone structural parameters in long bones and vertebrae (Wang et al., 2015; Kim et al., 2020) and lower bone formation following tooth extraction (Apaza Alccayhuaman et al., 2021). Consistently, conditional knock‐out of FasL in osteoblasts results in reduced femoral bone mass (Wang et al., 2015). There is thus a controversial picture regarding the impact of FasL on affecting bone mass, which may also affect the alveolar bone in periodontal health and disease.

Periodontitis, a chronic inflammatory disease affecting almost half of the adult population (Eke et al., 2012), is considered a major cause of tooth loss (Pihlstrom et al., 2005). Soluble FasL was found to be higher in gingival crevicular fluid obtained from patients with chronic periodontitis than in healthy controls (Dabiri et al., 2016) and the jawbone of human foetuses revealed the expression of Fas on both osteoblasts and osteoclasts and of Fas ligand on osteoblasts (Hatakeyama et al., 2000). *Porphyromonas gingivalis* can induce epithelial cell apoptosis through Fas–FasL activation of caspases (Brozovic et al., 2006), even though FasL polymorphism was not associated with severe chronic periodontitis (Wohlfahrt et al., 2006; Asgari et al., 2018). Considering that FasL affects the immune system (Griffith et al., 1995; Tsutsui et al., 1996; Nagata, 1997), the question arises whether FasL affects inflammatory osteolysis. Inflammatory osteolysis is an umbrella term that integrates all molecular and cellular events that culminate in bone loss (Mbalaviele et al., 2017; Gruber, 2019). Our aim was to discover the role of FasL during periodontitis‐mediated inflammatory osteolysis.

Mouse models allow us to study periodontal disease's pathogenesis and test new therapeutic approaches (Lin et al., 2021). The intended purpose of the ligature‐induced periodontitis model is to increase the local accumulation of microbial plaque and thereby enhance microbial‐mediated inflammation and bone loss (Marchesan et al., 2018; Lin et al., 2021). Bone loss is the hallmark of periodontal disease progression, increasing the distance between the cementum enamel junction (CEJ) and the alveolar bone crest (ABC) (Hienz et al., 2015). Harnessing the potential of micro‐computed tomography (μCT), 3D evaluation of the bone changes can be measured (Wilensky et al., 2005; Y. H. Wu et al., 2020; Catunda et al., 2021). Histological analysis can help describe and quantify alveolar bone osteolysis (Semenoff et al., 2008; de Molon et al., 2018). Implementing and refining established methods, the present study aimed to explore the periodontium of mice carrying the Fasl^*gld*^ mutation and the respective WT mice under physiological conditions and upon ligature‐induced periodontitis.

## 2 MATERIAL AND METHODS

### 2.1 Study design

The Medical University of Vienna Ethical Review Board for Animal Research approved the study protocol (GZ BMWFW‐66.009/0359‐V/3b/2018). The study was performed at the Department of Biomedical Research of the Medical University of Vienna following ARRIVE guidelines. Female and male mice homozygous for the Fasl^*gld*^ mutation (B6Smn.C3‐Fasl^*gld*^/J) were purchased from The Jackson Laboratory (Bar Harbour, ME, USA). C57BL/6 served as WT control. Animals were housed in the Medical University of Vienna, Institute of Biomedical Research, under specific‐pathogen‐free conditions. Fasl^*gld*^ mice and WT littermates underwent ligature placement at around 10–12 weeks. According to the animal welfare guidelines, the animals were maintained with free access to water and a diet based on cereal grains (Kilkenny et al., 2010).

### 2.2 Ligature‐induced periodontitis model

Ligature‐induced periodontitis was established according to Marchesan et al. (2018). All animals received ketamine 100 mg/kg (AniMedica, Senden, Erlangen, Germany) and xylazine hydrochloride 5 mg/kg (Bayer Austria, Vienna, Austria) by intramuscular injection. Mice were stabilized on a printed mouse bed to keep the mouth open (Marchesan et al., 2018). Using a printed ligature holder, a dual‐knotted 4‐0 silk ligature (Ethicon, Somerville, NJ, USA) of 2.5 mm length was placed between the upper first (M1) and second molar (M2). Based on the 10%–20% predicted ligature loss, the ligature was placed bilaterally (Marchesan et al., 2018). Mice were monitored and kept warm until they recovered from the anaesthesia. For pain relief, buprenorphine 0.06 mg/kg s.c. (Temgesic; Reckitt and Colman Pharm., Hull, UK) and piritramide in drinking water ad libitum were administered. For the first 72 h after surgery, a soft food consisting of pellets soaked in water was provided. Mice were euthanized on day 12, and only jaws where the ligature remained in situ were considered for analysis.

### 2.3 Micro‐CT analysis

**FIGURE 1:**
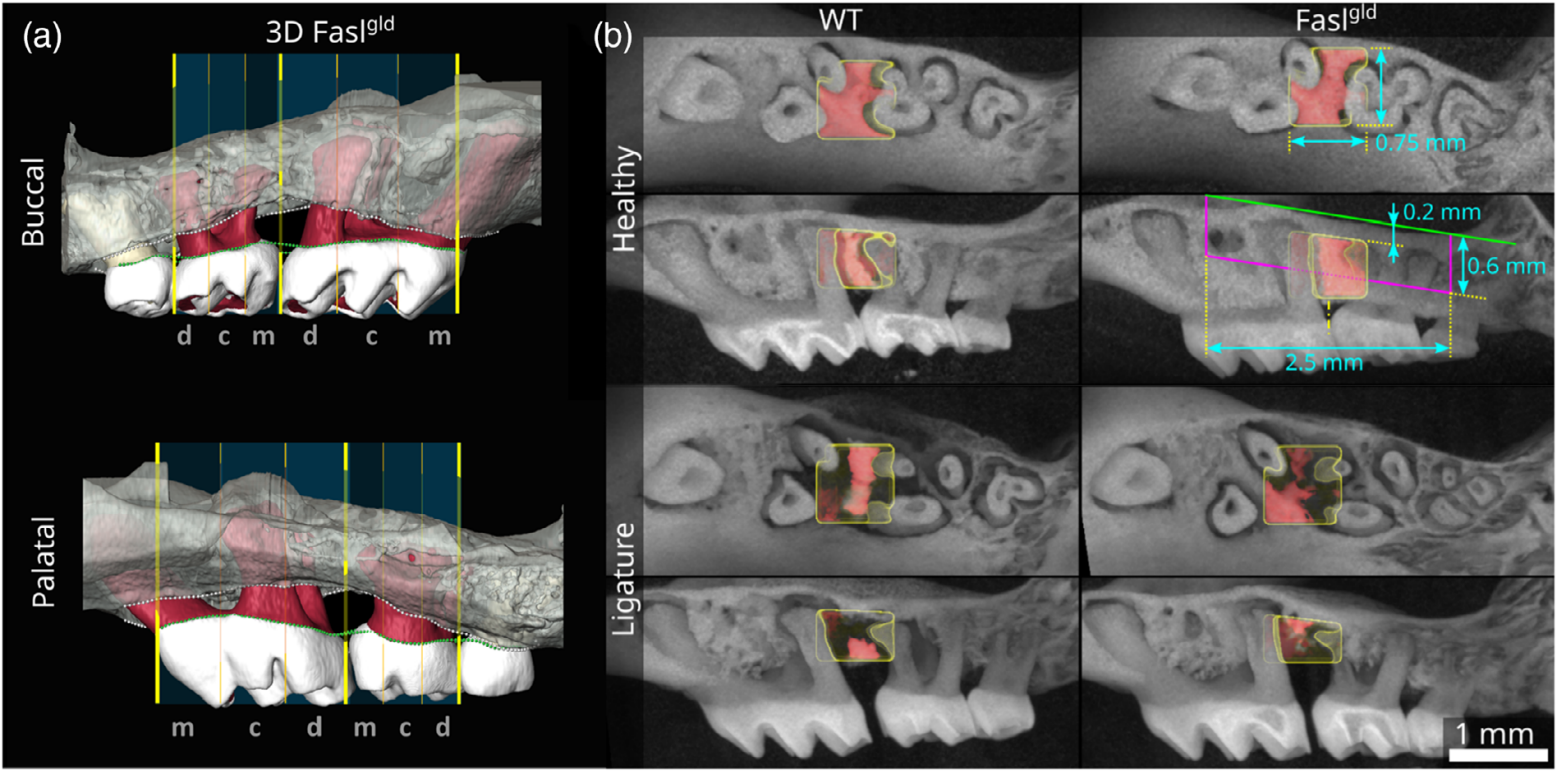
Linear and volumetric measurement in the region of interest. (a) The distance between cement enamel junction (CEJ) the alveolar bone crest (ABC) was measured by point‐based image registration. In the three‐dimensional (3D) isosurface of the wild‐type (WT) and Fasl^*gld*^ mice, B‐splines were placed connecting the CEJ (green knots) and the alveolar bone crest (white knots) on the buccal and palatal side. The first and second molars were divided into three parts: mesial (m), central (c), and distal (d). (b) An equally sized volume of interest (VOI) was set based on the ligature position between M1 and M2, for the healthy and the ligature‐induced periodontitis group with their respective WT and Fasl^*gld*^. Periodontal ligament space volume in healthy samples was measured in a 2.5‐mm‐wide region centred on the gap between M1 and M2 in the most apical 0.6 mm relative to the tip of the roots of M1 and M3.

The heads were fixed in 4% phosphate‐buffered formalin (Roti‐Histofix, Carl Roth, Karlsruhe, Germany). Micro‐CT scans (Scanco μCT 50; Scanco Medical AG, Bruttisellen, Switzerland) were taken at 90 kV/200 μA with an isotropic resolution of 8 μm and an integration time of 500 ms. Volume‐rendering and segmentation of the bone and teeth were done (Amira 6.1.1; Thermo Fisher Scientific, Waltham, MA, USA) (Video S1). For linear measurements of the CEJ–ABC, a set of B‐splines (equidistant knots along the CEJ and ABC) were placed at the buccal and palatal sites of the molars (M1–M3). Using linear regression, the distance was measured every 50 μm perpendicular to a plane fitted to the CEJ. The mesial, central, and distal part of the teeth were distinguished (Figure 1a). For volumetric analysis (Definiens Developer XD 2.7; Definiens AG, Munich, Germany), the segmented files were aligned using Fiji software (Schindelin et al., 2012). The volume of interest (VOI) for measurement of the bone was set between M1 and M2. A virtual plane was created from the tip of the roots of M1 to M3. The bone VOI began 0.2 mm coronal of this plane and extended to the alveolar crest. In buccal–palatal direction, the VOI was limited to the tooth width. In mesial–distal direction, the VOI is constrained by surface extension, which limits the VOI to the space between the roots (Figure 1b, red bone). Bone volume fraction (BV/TV) was measured in this VOI. The periodontal ligament space volume (PLS.V) was measured in a region of 2.5 mm length in the mesial–distal direction, centred on the gap between M1 and M2 and extending 0.6 mm coronal of the virtual plane at the root tips (Figure 1b, magenta line). Because of the heavy resorption of bone in the ligature group, measurement of PLS.V in the same VOI was not possible. Instead, PLS.V was measured in the bone VOI for those samples. The tooth volumes were measured (M1.V and M2.V) unlimited by any VOI.

### 2.4 Histological analysis

The samples were dehydrated with ascending alcohol grades and embedded in a light‐curing resin (Technovit 7200 VLC + BPO; Kulzer & Co., Wehrheim, Germany). The cutting plane was planned by μCT. Blocks were processed using a cutting and grinding equipment (Exakt Apparatebau, Norderstedt, Germany). Thin ground sections were stained with Levai–Laczko dye (Morphisto GmbH, Frankfurt, Germany) (Donath & Breuner, 1982). The sections were scanned using an Olympus BX61VS digital virtual microscopy system (DotSlide 2.4, Olympus, Tokyo, Japan) at ×20 magnification, resulting in 0.32 μm per pixel. Using a 25‐μm grid, the eroded bone and root surface between M1 and M2 was quantified. The distance between the CEJ and the inter‐proximal alveolar bone was also measured.

### 2.5 Statistical analysis

Statistical analysis was based on the multilevel data obtained from the μCT analysis. Descriptive statistics, unpaired Student's *t*‐test, and plotting of the data were performed by Prism 9 (GraphPad Software, San Diego, CA, USA). Significance was set at *p* < .05. To study the potential influence of FasL on the healthy and ligature‐induced periodontitis at the tooth level, a linear mixed regression model was used, where the strains (WT or Fasl^*gld*^), location (buccal or palatal), teeth (M1 or M2), and position (mesial, central, or distal) were fitted as fixed effects. The random effect was the mouse ID. This analysis respects the dependence structure of the data and was performed using the R version 4.0.2 (Team, 2022). The sample size calculation was performed using G*power 4 (Düsseldorf, Germany) based on data from previous studies (Marchesan et al., 2018). Considering a bone loss of 0.49 mm with an SD of 0.08 and expecting a 30% of variation between the WT and the Fasl^*gld*^ groups, with a 90% power and type I error rate of 5%, we estimated a sample size of 16 mice per strain. The primary outcome of the ligature model was the distance between CEJ and ABC.

## 3 RESULTS

### 3.1 Alveolar bone crest level under healthy conditions and ligature‐induced periodontitis

**FIGURE 2:**
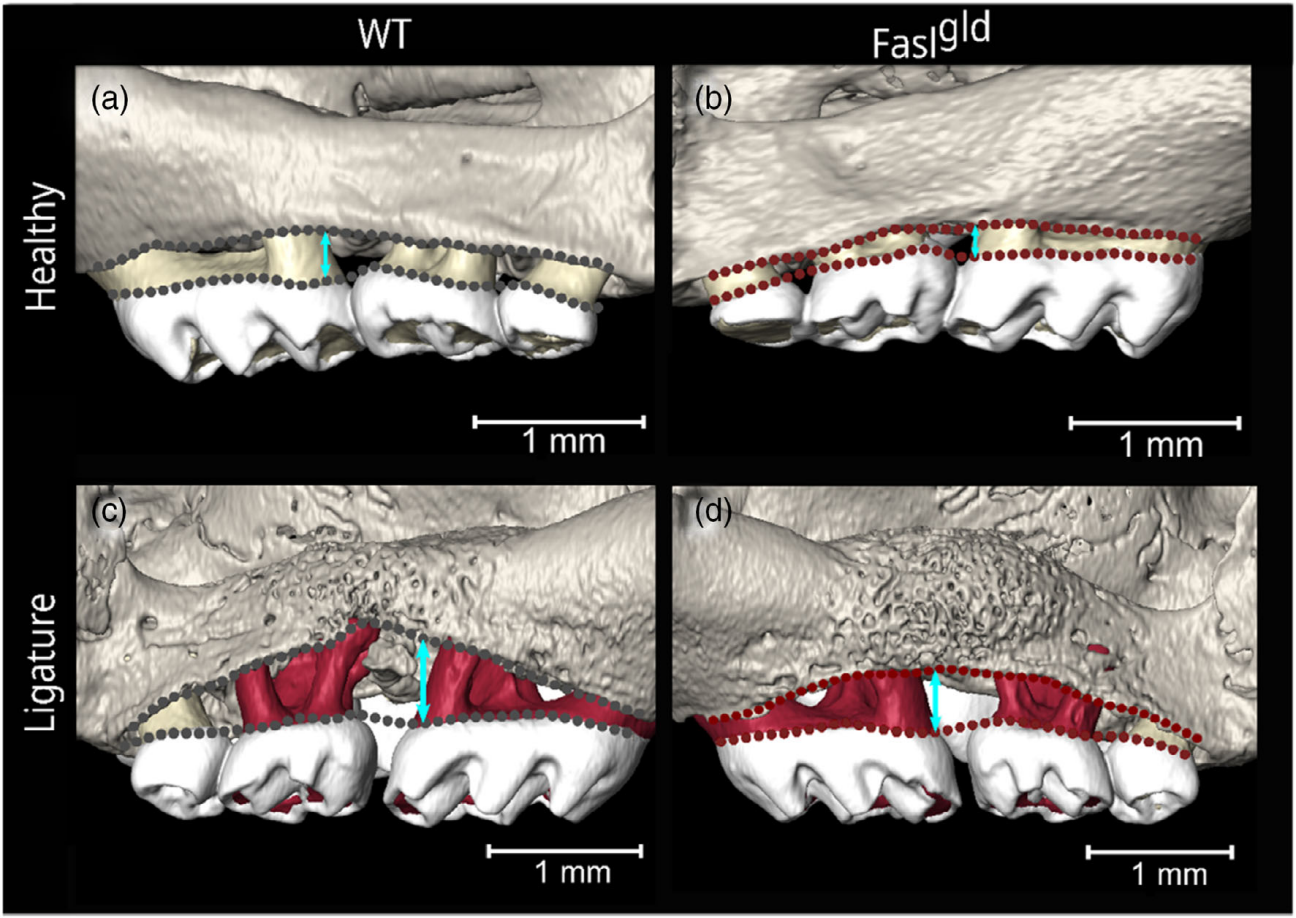
Linear measurements of CEJ–ABC distance. Representative 3D images of the buccal side showing a higher CEJ–ABC in the WT (a) compared to the Fasl^*gld*^ (b) under healthy conditions. After 12 days of ligature‐induced periodontitis, both WT (c) and Fasl^*gld*^ mice (d) presented substantial bone loss (representative double arrows show the distance between CEJ and ABC (b‐splines) and the porous structure of the compensatory fast‐growing woven bone as revealed by histology of Figure 6. ABC, alveolar bone crest; CEJ, cementum enamel junction; WT, wild‐type

**FIGURE 3:**
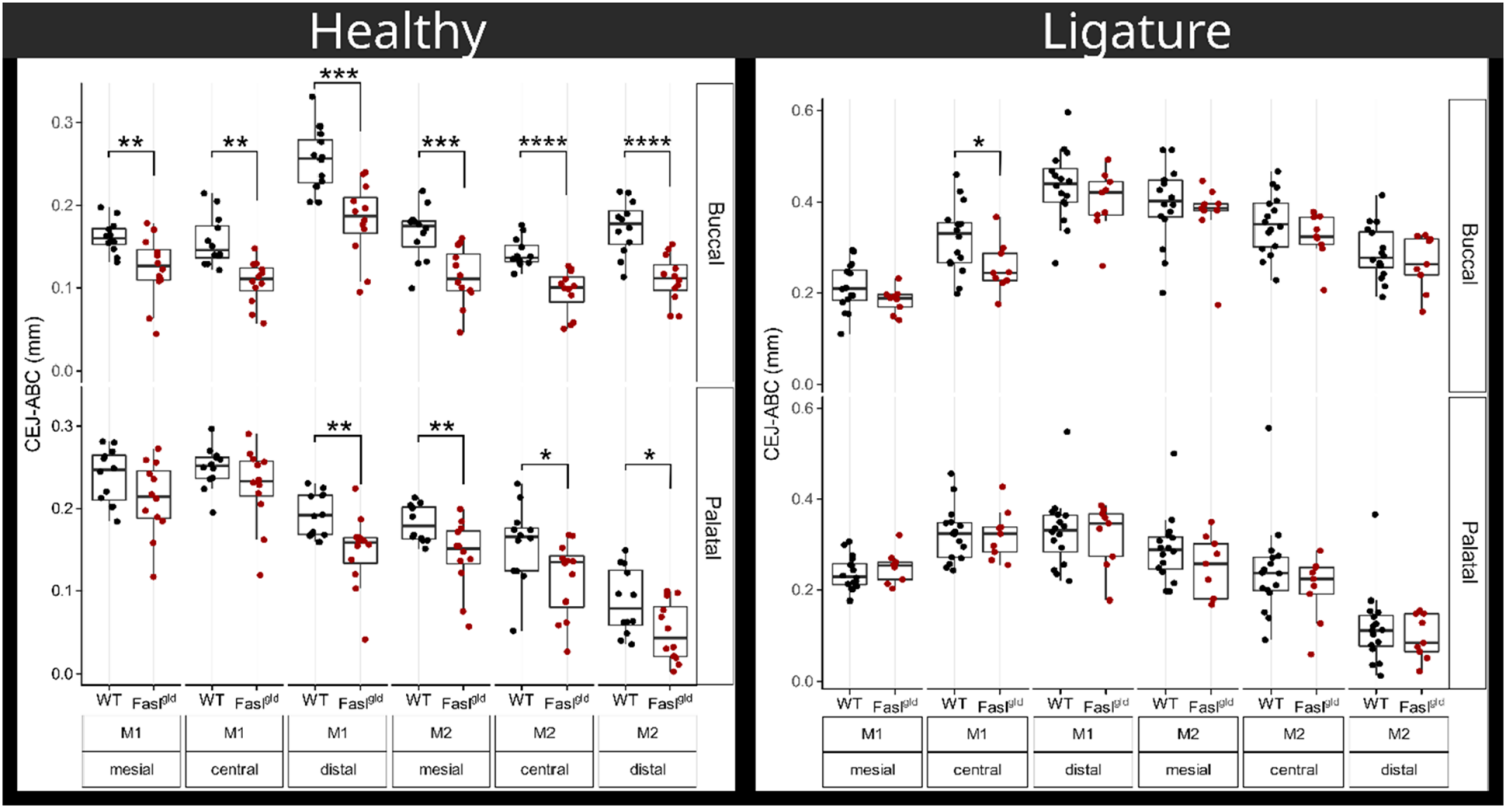
Linear measurements of the CEJ–ABC distance. Under healthy condition, CEJ–ABC was smaller in Fasl^*gld*^ mice compared to WT mice in the buccal and the palatal aspects. After ligature‐induced periodontitis, CEJ–ABC failed to reach the level of significance when comparing WT and Fasl^*gld*^ mice. The strain (WT/Fasl^*gld*^), teeth (M1/M2), and position (mesial, central, and distal) are displayed on the *x*‐axis. For the healthy animals, each dot represents the left and right side of the maxilla for WT (*n* = 12) and Fasl^*gld*^ (*n* = 12). For ligature‐induced periodontitis, each dot represents the location with remaining ligatures in WT (*n* = 16) and Fasl^*gld*^ (*n* = 9). *p < .05; **p < .01; ***p < .001; ****p < .0001 WT versus Fasl^*gld*^. ABC, alveolar bone crest; CEJ, cementum enamel junction; WT, wild‐type

To understand how FasL affects the maxillary bone under healthy conditions, the distance between CEJ and ABC was measured in WT (*n* = 6) and Fasl^*gld*^ (*n* = 6) female mice. The alveolar crest level was significantly higher in Fasl^*gld*^ than in WT mice (Figure 2a,b), particularly in the buccal M1 distal position (*p =* .0003), the M2 mesial (*p* = .0009), and the M2 central and distal part (*p = <*.0001) (Figure 3, healthy). In the palatal side, this effect was less pronounced (Figure 3, palatal). There were no clinically relevant differences between locations (left or right) (Table S1).

Under healthy conditions, the alveolar crest level was significantly influenced by the lack of FasL (*p =* .0107). Nevertheless, under inflammatory osteolysis, the CEJ–ABC distance in the WT (*n* = 14) and Fasl^*gld*^ (*n* = 8) mice (Figure 3, ligature), the statistical analysis revealed no impact of the mouse phenotype on CEJ–ABC. However, considering the original ABC level, we have calculated the average net loss by subtracting the median CEJ–ABC of mice with and without ligatures. Fasl^*gld*^ mice lost only marginally more buccal bone (0.20 and 0.23 mm; M1 distal and M2 mesial) than WT animals (0.17 and 0.20 mm, respectively). Also, in the palatal aspect, Fasl^*gld*^ lost only slightly more bone (0.17 mm; M1 distal) than WT animals (0.12 mm; M1 distal; Table S2). Descriptive statistics showed no differences in CEJ–ABC regarding gender and location (Tables S3 and S4).

### 3.2 Alveolar bone volume fraction under healthy conditions and ligature‐induced periodontitis

**FIGURE 4:**
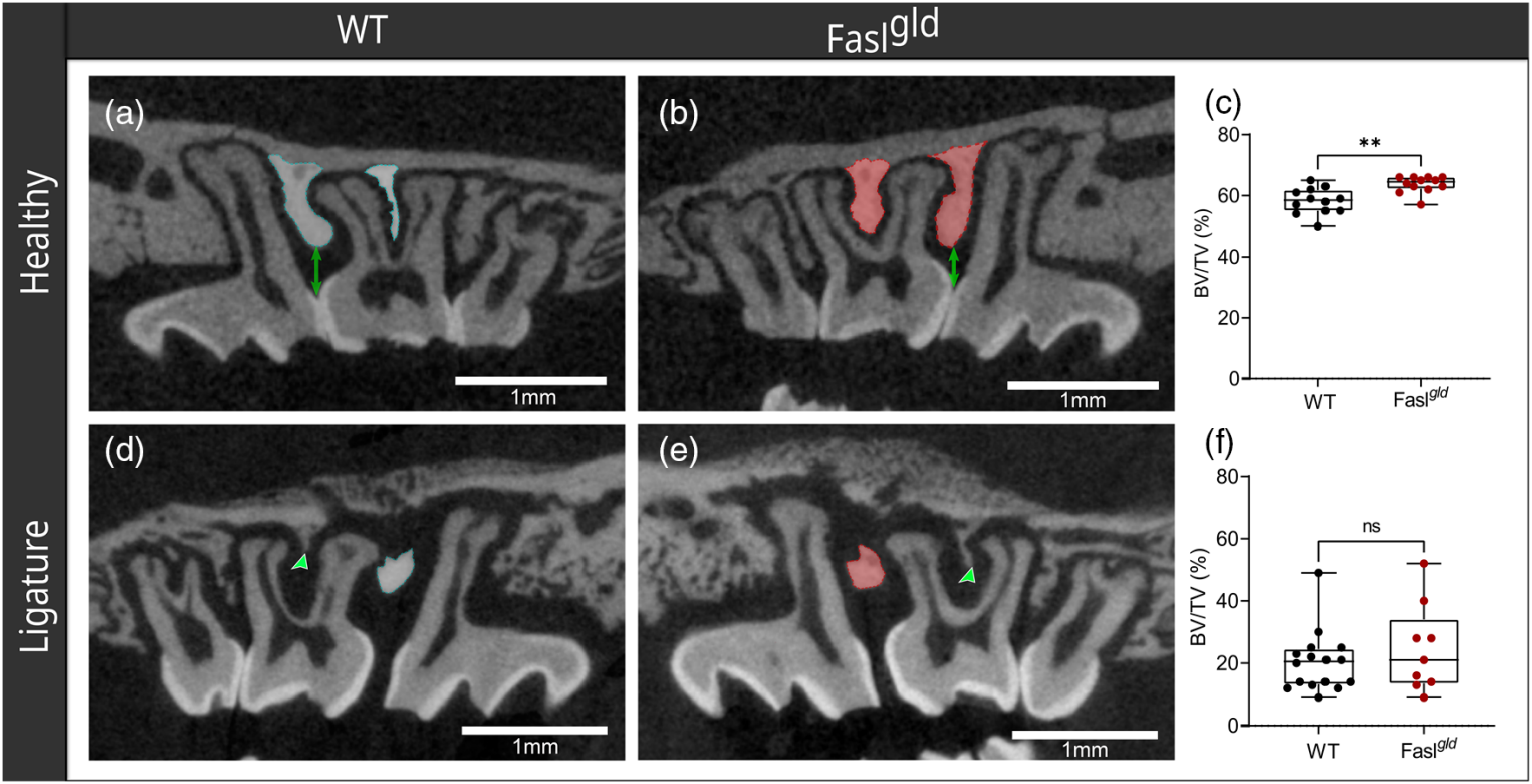
Volumetric measurements of the region of interest. Under healthy conditions, representative μCT images revealed that WT (a, cyan outline) have less inter‐proximal bone (between M1 and M2) and inter‐radicular septum of the M2 compared to Fasl^*gld*^ (b, red outline). The green double arrows show the distance between ABC and the contact point. The respective difference in BV/TV is shown in the boxplots with **p < .01 (c). Under ligature‐induced periodontitis, almost the entire inter‐proximal (cyan and red outline) and inter‐radicular (green arrows) bones were lost in WT (d) and Fasl^*gld*^ (e) mice. There were no significant (ns) differences regarding the BV/TV (f). ABC, alveolar bone crest; WT, wild‐type; μCT, micro‐computed tomography

Next, the BV/TV was analysed. Under healthy conditions, the BV/TV of WT (58.17 ± 4.3%) was significantly lower than in Fasl^*gld*^ (63.67 ± 2.7%) mice (*p* = .001, Figure 4, healthy row). Ligature‐induced periodontitis caused strong bone loss in both WT (20.25 ± 9.7%) and Fasl^*gld*^ (24.56 ± 14.09%) mice, which failed to reach the statistical significance (*p* = .37, Figure 4, row ligature). The calculated loss of bone volume was comparable between both mouse strains. Taken together, Fasl^*gld*^ mice displayed more alveolar bone mass under healthy conditions than WT mice; however, the inflammatory osteolysis is similarly affected in both strains.

### 3.3 Volume of the periodontal ligament under healthy and ligature‐induced periodontitis

**FIGURE 5:**
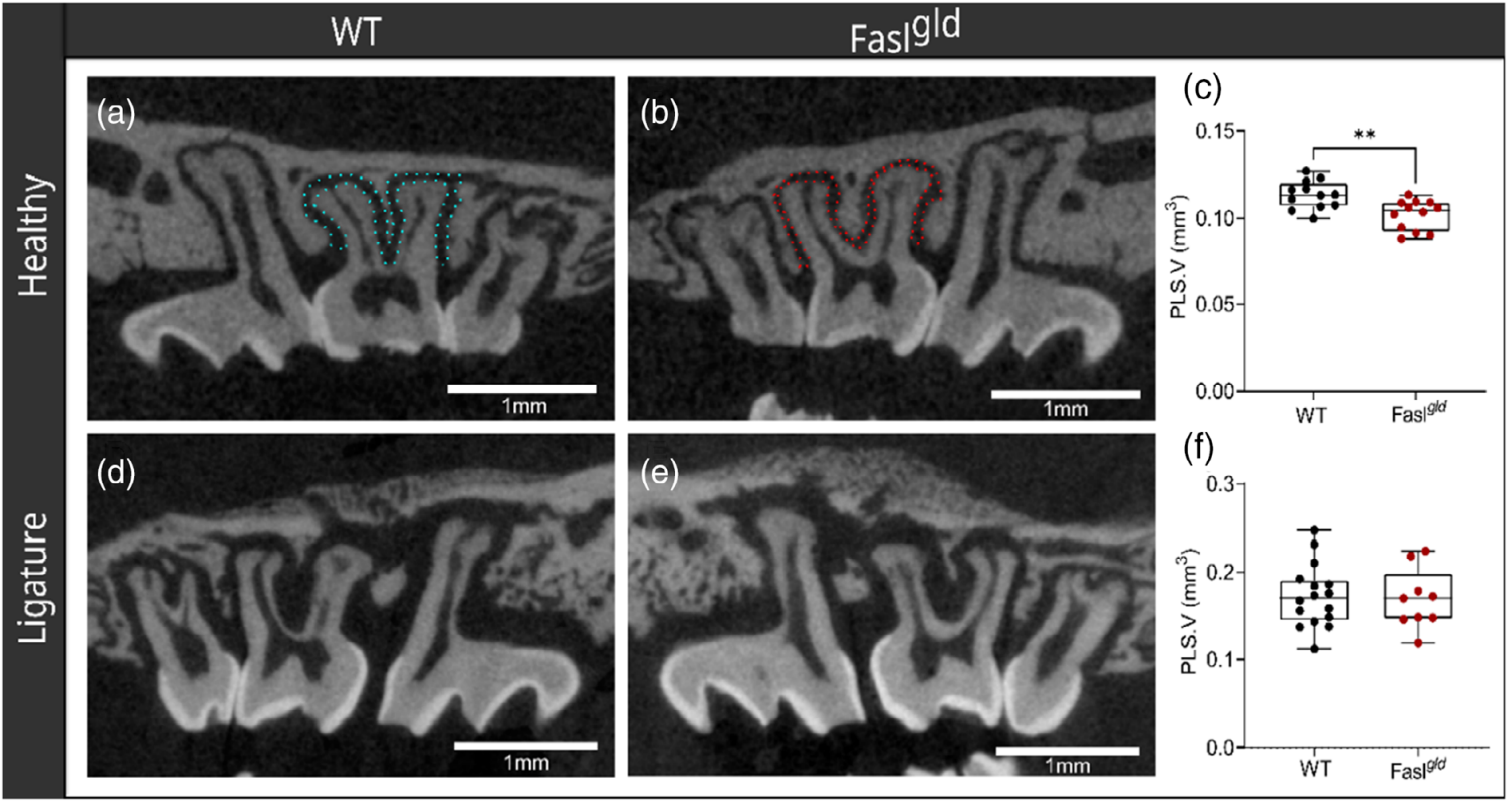
Volumetric measurements of the periodontal ligament space. Under healthy conditions, representative μCT images revealed that WT (a, cyan outline) had a higher periodontal ligament volume (PLS.V) compared to Fasl^*gld*^ (b, red outline), which was confirmed by quantitative measures with **p < .01 (c). Under the ligature‐induced periodontitis, PLS.V considerably expanded in both strains at the inter‐proximal bone and inter‐radicular septum (d, e). The boxplots did not show significant difference (ns) for WT and Fasl^*gld*^ (f). WT, wild‐type; μCT, micro‐computed tomography

Periodontal ligament volume (PLS.V) under healthy conditions in WT (0.11 ± 0.008 mm^3^) mice was higher than in Fasl^*gld*^ (0.10 ± 0.009 mm^3^) mice (*p =* .003; Figure 5, healthy row). After ligature‐induced periodontitis, there was no statistically significant difference (*p =* .82, Figure 5, ligature row) between WT (0.17 ± 0.04 mm^3^) and Fasl^*gld*^ (0.17 ± 0.03 mm^3^) mice. The tooth volume was not affected in Fasl^*gld*^ mice compared to their WT littermates (Figure S1). Furthermore, after ligature placement, the inter‐dental space became wider in WT (0.07 ± 0.06 mm) and Fasl^*gld*^ (0.05 ± 0.06 mm) mice; however, there was no significant difference (*p* = .5) between the strains.

### 3.4 Histological analyses

**FIGURE 6:**
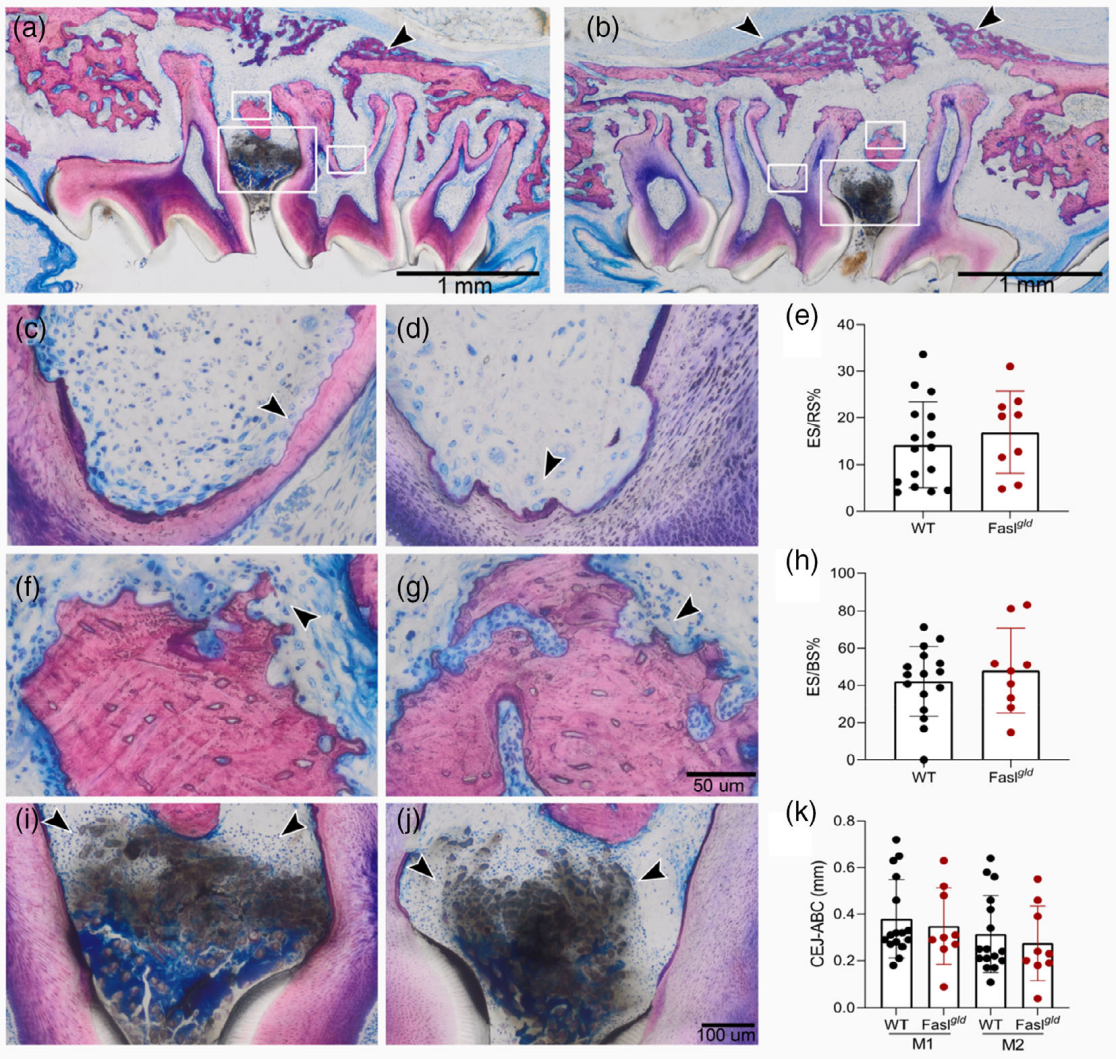
Representative histological images of WT and Fasl^*gld*^ mice. Low‐magnification overviews revealed the morphological features of inflammatory osteolysis caused by the ligatures that are visible between M1 and M2 in WT (a) and Fasl^*gld*^ (b) mice. Arrows point towards the signs of bone formation originating from the periosteum to compensate the severe bone resorption caused by the inflammatory osteolysis. The higher magnification images display the eroded root surface in the furcation in WT (c) and Fasl^*gld*^ mice (d). Also, severe bone resorption of the ABC in WT (f) and Fasl^*gld*^ (g) is clearly visible. The silk ligature provoked an obvious inflammatory infiltrate (arrows pointing) for WT (i) and Fasl^*gld*^ mice (j). There was no difference between the strains regarding the percentage of the eroded root surface (ER/RS%) (e) and at the eroded bone surface (ER/BS%) (h). The CEJ to the inter‐proximal ABC length measured at M1 and M2 also showed no significance between the two strains (k). ABC, alveolar bone crest; CEJ, cementum enamel junction; WT, wild‐type

Finally, we assessed the morphological features of inflammatory osteolysis by histological analysis. Signs of bone resorption came along with fast‐growing woven bone originating from the periosteum in both strains (arrows pointing in Figure 6a,b; see also Figure S2). Resorption of dentin and cementum was present in WT and Fasl^*gld*^ mice (arrows pointing in Figure 6c,d). Surface erosion was comparable between WT (42.23 ± 18.7%) and Fasl^*gld*^ (48.02 ± 22.8%) mice (Figure 6e,h). To complement the μCT measurements, the CEJ–ABC distance was determined at the inter‐proximal bone of M1 and M2. Again, there was no significant differences between WT (0.38 ± 0.17; 0.31 ± 0.17 mm) and Fasl^*gld*^ (0.35 ± 0.16; 0.28 ± 0.16 mm) mice (Figure 6k). On a descriptive level, the junctional epithelium of the mesial part of M1 was not obviously affected by the Fasl^*gld*^ mutation (Figure S3).

## 4 DISCUSSION

The present study supports existing evidence that Fasl^*gld*^ mice experience denser bones, less bone loss after ovariectomy, and more trabecular bone after bone marrow ablation than WT controls (Katavic, Lukic, et al., 2003). Moreover, in Fasl^*gld*^ mice more ectopic bone and cartilage formation was induced upon injection of natural bone morphogenetic protein (BMP) and recombinant BMP‐2 compared to WT controls (Mori et al., 1997; Katavic, Grcevic, et al., 2003). These findings do not necessarily reflect the cranial tissues because of the type of ossification (Berendsen & Olsen, 2015) and the biomechanical situation (McElhaney et al., 1970). Nevertheless, jawbone of human foetuses has revealed the expression of Fas on both osteoblasts and osteoclasts and of Fas ligand on osteoblasts (Hatakeyama et al., 2000). To understand the impact of FasL on the periodontal tissues, we have analysed the alveolar bone in Fasl^*gld*^ mice and the respective WT controls under healthy conditions and ligature‐induced periodontitis.

Our first main finding is that Fasl^*gld*^ mice display a significantly higher alveolar bone level and bone volume compared to the WT mice under healthy conditions. The difference was somewhat stronger on the buccal side compared to the palatal side. Particularly, we found a substantial difference at the distal part of M1 and M2 based on advanced image processing and algorithms for tissue segmentation. Consistently, the volume of the periodontal ligament was lower in Fasl^*gld*^ mice than in WT mice. Indirect support for our observations comes from 24‐day‐old Fasl^*gld*^ mice, which displayed increased bone volume and trabecular thickness (Svandova et al., 2019). The underlying cellular mechanism may involve an anabolic shift in which osteoblastic bone formation exceeds osteoclastic bone resorption (Katavic, Lukic, et al., 2003). Future research could therefore consider lineage‐specific knock‐out mice where Fasl^*gld*^ is exclusively expressed in osteoblast, osteocytes, or osteoclast. Moreover, contrasting findings that FasL is required to reach adequate bone mass in the axial and appendicular skeleton leave us with uncertainty (Wang et al., 2015; Kim et al., 2020).

Knowing why healthy Fasl^*gld*^ mice have more periodontal bone than WT littermates might involve the effect of lymphocytes—all of which are known to modulate osteoblast and osteoclast formation, activity, and death (Mbalaviele et al., 2017; Gruber, 2019). Thus, the generalized lymphoproliferative disorder caused by the Fasl^*gld*^ mutation could indirectly affect bone turnover. At the level of the oral cavity, lymphoproliferative disorder can occur in various ways, most commonly as lymphoid lesions with extranodal externalization (Castellarin et al., 2010); nothing similar was observed in the oral histology of our mice. Fasl^*gld*^ mice failed to generate oral diseases that could be linked to lymphoproliferative disorder. It can thus be speculated that the possible impact of lymphoproliferative disorder in Fasl^*gld*^ mutations on the periodontium was bone anabolic.

Contrasting to our Fasl^*gld*^ mice study, in vitro studies showed that FasL reduced the WNT‐antagonist sclerostin in IDG‐SW3 osteocytic cells, suggesting an anabolic activity of the ligand (Kratochvilova et al., 2021). Nevertheless, the WNT pathways plays a role in regulating the stem cell niche at the interface between tooth and oral epithelia (Yuan et al., 2020), and activation of WNT signalling promotes FasL expression in periodontal ligament cells (Yu et al., 2019). In general, however, the evidence for a functional link between FasL and WNT signalling is low. Considering the importance of TGF‐β in bone biology (Crane & Cao, 2014), TGF‐β1 and FasL can act synergitically in dendritic cell activation (Qiu et al., 2017). Moreover, the FasL‐mediated apoptosis pathway is regulated by TGF‐β3 signalling, which is essential for palatal fusion during craniofacial development (Huang et al., 2011). TGF‐β1 can also affect the sensitivity of lung epithelial cells to FasL‐mediated apoptosis (Bai et al., 2011). Thus, it is possible that, in our model, impaired FasL signalling has increased the responsiveness of bone cells to growth factors and other signalling molecules.

The second main finding is that, upon ligature‐induced inflammatory osteolysis, the CEJ–ABC distance and the volume of the remaining alveolar bone were similar in WT and Fasl^*gld*^ mice. It can be concluded that Fasl^*gld*^ does not substantially prevent or enhance inflammatory osteolysis, also when considering that healthy Fasl^*gld*^ mice originally had more alveolar bone than the WT mice. These findings are in line with other mouse models showing no effects of FasL on induced pneumococcal meningitis (Paul et al., 2004) and chemically induced colitis (Reardon et al., 2008). Moreover, there is controversial in vitro evidence that osteoclasts are targets of FasL‐stimulated apoptosis (X. Wu et al., 2005), while recombinant FasL enhanced osteoclast differentiation involving the expression of inflammatory cytokines (Park et al., 2005). In general, FasL can reduce inflammation by triggering apoptosis of inflammatory cells but exhibits a pro‐inflammatory activity independent of its ability to mediate immune privilege (O'Connell, 2000). The effects of FasL are thus heterogenous and we must carefully interpret the role of FasL in modulating periodontal inflammatory osteolysis.

The clinical relevance of our findings remains at the level of speculation, but we can at least conclude that a non‐functional FasL is beneficial for the alveolar bone in a healthy periodontium and that FasL is not the main factor when it comes to the catabolic events driving inflammatory osteolysis. It seems that FasL is weak if at all a candidate molecule to be targeted by pharmacological therapy (Risso et al., 2022). Moreover and even though FasL was higher in gingival crevicular fluid obtained from patients with chronic periodontitis than in healthy patients (Dabiri et al., 2016), our mouse model suggests that the higher expression of FasL is presumably a consequence of the periodontitis but not a major driver of inflammatory osteolysis. What needs to be tested in future studies is the possibility that pharmacological blocking of FasL supports bone regeneration, although our recent tooth extraction model on Fasl^*gld*^ mice points in another direction (Apaza Alccayhuaman et al., 2021).

The present study has various limitations. The major limitation is that the study is descriptive, and the molecular and cellular mechanisms to explain the higher bone mass displayed in the Fasl^*gld*^ remain unclear. Future studies may consider a specific knock‐out mouse to discover the mechanisms involved and consider the bone remodelling parameters regarding the osteoblast–osteoclast differentiation, activity, and cell fate. Moreover, DMP1‐Fasl^*gld*^ conditional knock‐out mice would show us the importance of osteocytes in regulating bone remodelling and inflammatory osteolysis. Another limitation was that the ligature slipped off before the expected period; thus the animals were excluded from the analysis and we were left with an unbalanced sample requiring a mixed regression model. The histology from the healthy periodontium is not shown here but high‐resolution μCT images reflect the healthy phenotype. Also, Technovit 7200 embedding is not ideal to perform phenotyping of neutrophils and other cells of the innate immunity as well as staining for the osteoclast marker tartrate‐resistant acid phosphatase. We can, however, identify the resorption lacunae, and occasionally we see multinucleated cells that are most likely osteoclasts. In conclusion, we have discovered that in healthy conditions, Fasl^*gld*^ mice have more alveolar bone compared to their WT littermates. Concerning inflammatory osteolysis, Fasl^*gld*^ and WT mice both showed similar levels of remaining alveolar bone, suggesting that FasL is not critically involved in inflammatory osteolysis.

## CONFLICT OF INTEREST

The authors declare no conflicts of interest related to this study.

## Supporting information


**Table S1.** Descriptive statistics for cement enamel junction–alveolar bone crest distance per location under healthy conditions.
**Table S2.** Bone level changes in wild‐type and Fasl^*gld*^ mice under healthy and ligature‐induced periodontitis.
**Table S3.** Descriptive statistics for length per sex under ligature‐induced periodontitis.
**Table S4.** Descriptive statistics for length per location under ligature‐induced periodontitis.
**Figure S1.** Under healthy conditions and upon ligature‐induced periodontitis, there were no differences regarding the volume of the molars (M1 and M2) for both strains.
**Figure S2.** Representative histological sections of wild‐type and Fasl^*gld*^ mice displayed fast‐growing woven bone originating from the periosteum.
**Figure S3.** Representative histological sections of wild‐type and Fasl^*gld*^ mice displayed the junctional epithelium. Arrowheads point towards the cementum enamel junction. The picture represents the mesial part of the first molar.

**Video S1.** Volume rendering of the periodontal structure under physiological and inflammatory condition.

## Data Availability

The data that support the findings of this study are available from the corresponding author upon reasonable request.
