# Supplementary material for: FasL is a catabolic factor in alveolar bone homeostasis
Source: J Clin Periodontol. 2022 Nov 25;50(3):396–405. doi: 10.1111/jcpe.13750 (PMC10946845; doi:10.1111/jcpe.13750)
Supplement: Supplementary file 1 — Table S1. Descriptive statistics for cement enamel junction–alveolar bone crest distance per location under healthy conditions. Table S2. Bone level changes in wild‐type and Fasl gld mice under healthy and ligature‐induced periodontitis. Table S3. Descriptive statistics for length per sex under ligature‐induced periodontitis. Table S4. Descriptive statistics for length per location under ligature‐induced periodontitis. Figure S1. Under healthy conditions and upon ligature‐induced periodontitis, there were no differences regarding the volume of the molars (M1 and M2) for both strains. Figure S2. Representative histological sections of wild‐type and Fasl gld mice displayed fast‐growing woven bone originating from the periosteum. Figure S3. Representative histological sections of wild‐type and Fasl gld mice displayed the junctional epithelium. Arrowheads point towards the cementum enamel junction. The picture represents the mesial part of the first molar. [file JCPE-50-396-s001.docx]

**FasL is a catabolic factor in alveolar bone homeostasis**

Karol Alí Apaza Alccayhuaman (1, 2), Patrick Heimel (2, 3, 4), Jung Seok Lee (1,5), Stefan Tangl (2, 4), Ulrike Kuchler (6), Julie Marchesan (7), Layla Panahipour (1), Stefan Lettner (2) Eva Matalová (8), Reinhard Gruber (1, 4, 9)

1. Department of Oral Biology, University Clinic of Dentistry, Medical University of Vienna, Austria
2. Karl Donath Laboratory for Hard Tissue and Biomaterial Research, University Clinic of Dentistry, Medical University of Vienna, Austria
3. Ludwig Boltzmann Institute for Traumatology, The Research Center in Cooperation with AUVA, Vienna, Austria.
4. Austrian Cluster for Tissue Regeneration, Vienna, Austria
5. Department of Periodontology, Research Institute for Periodontal Regeneration, College of Dentistry, Yonsei University, Seoul, Republic of Korea
6. Department of Oral Surgery, University Clinic of Dentistry, Medical University of Vienna, Austria
7. Division of Comprehensive Oral Health, Adams School of Dentistry, University of North Carolina at Chapel Hill, North Carolina, USA
8. Institute of Animal Physiology and Genetics, Czech Academy of Sciences, Brno, Czech Republic
9. Department of Periodontology, School of Dental Medicine, University of Bern, Switzerland

**Supplemental Tables**

| CEJ-ABC (mm) | | | | | | | | |
| --- | --- | --- | --- | --- | --- | --- | --- | --- |
| Strain | Side | Mean | SD | Min | Q1 | Median | Q3 | Max |
| WT | Left | 0.1941 | 0.05397 | 0.0462 | 0.1774 | 0.1962 | 0.2234 | 0.3008 |
|  | Right | 0.1900 | 0.05776 | 0.0338 | 0.1590 | 0.1875 | 0.2311 | 0.3311 |
| Fasl^gld^ | Left | 0.1592 | 0.05408 | 0.0423 | 0.1314 | 0.1592 | 0.1878 | 0.2965 |
|  | Right | 0.1519 | 0.05915 | 0.0292 | 0.1106 | 0.1498 | 0.1832 | 0.2813 |
| All | Left | 0.1766 | 0.05663 | 0.0423 | 0.1400 | 0.1818 | 0.2156 | 0.3008 |
|  | Right | 0.1710 | 0.06133 | 0.0292 | 0.1396 | 0.1663 | 0.2109 | 0.3311 |

Supplemental Table S1. Descriptive statistics for CEJ-ABC distance per location under healthy conditions.

*Note: SD: standard deviation; Q1: quartil 1; Q3: quartil 3*

Supplemental Table S2. Bone level changes in the WT and Fasl^gld^ mice under healthy and ligature-induced periodontitis.

| CEJ-ABC (mm) | | | | | | | | |
| --- | --- | --- | --- | --- | --- | --- | --- | --- |
| Strain | **WT** | | | | **Fasl^gld^** | | | |
|  | **Ligature** | | **Healthy** | | **Ligature** | | **Healthy** | |
|  | **dM1** | **mM2** | **dM1** | **mM2** | **dM1** | **mM2** | **dM1** | **mM2** |
|  | 0.36 | 0.40 | 0.27 | 0.19 | 0.45 | 0.39 | 0.25 | 0.14 |
|  | 0.52 | 0.37 | 0.22 | 0.20 | 0.37 | 0.40 | 0.24 | 0.18 |
|  | 0.60 | 0.52 | 0.29 | 0.13 | 0.46 | 0.42 | 0.20 | 0.13 |
|  | 0.34 | 0.27 | 0.24 | 0.18 | 0.43 | 0.38 | 0.18 | 0.13 |
|  | 0.27 | 0.20 | 0.27 | 0.16 | 0.49 | 0.40 | 0.20 | 0.13 |
|  | 0.45 | 0.46 | 0.25 | 0.20 | 0.26 | 0.17 | 0.22 | 0.15 |
|  | 0.46 | 0.45 | 0.24 | 0.20 | 0.42 | 0.45 | 0.14 | 0.11 |
|  | 0.40 | 0.30 | 0.29 | 0.22 | 0.36 | 0.36 | 0.13 | 0.09 |
|  | 0.41 | 0.36 | 0.22 | 0.16 | 0.38 | 0.38 | 0.21 | 0.19 |
|  | 0.51 | 0.52 | 0.27 | 0.20 |  |  | 0.22 | 0.16 |
|  | 0.42 | 0.44 | 0.33 | 0.20 |  |  | 0.20 | 0.18 |
|  | 0.45 | 0.39 | 0.30 | 0.23 |  |  | 0.25 | 0.17 |
|  | 0.49 | 0.45 |  |  |  |  |  |  |
|  | 0.40 | 0.38 |  |  |  |  |  |  |
|  | 0.47 | 0.41 |  |  |  |  |  |  |
|  | 0.44 | 0.43 |  |  |  |  |  |  |
| Buccal mean | 0.44 | 0.40 | 0.27 | 0.19 | 0.40 | 0.37 | 0.20 | 0.15 |
| ∆ means | 0.17 | 0.20 |  |  | 0.20 | 0.23 |  |  |

Supplemental Table S3: Descriptive statistics for length per sex under ligature-induced periodontitis

| CEJ-ABC (mm) | | | | | | | |
| --- | --- | --- | --- | --- | --- | --- | --- |
| Sex | Mean | SD | Min | Q1 | Median | Q3 | Max |
| Female | 0.2621 | 0.1311 | −0.0412 | 0.1904 | 0.2656 | 0.3502 | 0.5964 |
| Male | 0.2584 | 0.1068 | 0.0068 | 0.1964 | 0.2553 | 0.3403 | 0.4917 |

*Note: SD: standard deviation; Q1: quartil 1; Q3: quartil 3*

Supplemental Table S4. Descriptive statistics for length per location under ligature-induced periodontitis

| CEJ-ABC (mm) | | | | | | | | |
| --- | --- | --- | --- | --- | --- | --- | --- | --- |
| Strain | Side | Mean | SD | Min | Q_1_ | Median | Q_3_ | Max |
| WT | Left | 0*.*2648 | 0*.*1171 | −0*.*0121 | 0*.*2015 | 0*.*2631 | 0*.*3451 | 0*.*5569 |
|  | Right | 0*.*2720 | 0*.*1310 | −0*.*0298 | 0*.*2004 | 0*.*2754 | 0*.*3563 | 0*.*5964 |
| Fasl^gld^ | Left | 0*.*2468 | 0*.*1153 | −0*.*0222 | 0*.*1738 | 0*.*2521 | 0*.*3354 | 0*.*4941 |
|  | Right | 0*.*2454 | 0*.*1160 | −0*.*0412 | 0*.*1914 | 0*.*2509 | 0*.*3271 | 0*.*4590 |
| All | Left | 0*.*2583 | 0*.*1164 | −0*.*0222 | 0*.*1894 | 0*.*2564 | 0*.*3385 | 0*.*5569 |
|  | Right | 0*.*2625 | 0*.*1262 | −0*.*0412 | 0*.*1969 | 0*.*2643 | 0*.*3516 | 0*.*5964 |

*Note: SD: standard deviation; Q1: quartil 1; Q3: quartil 3*

**Supplemental Figures**


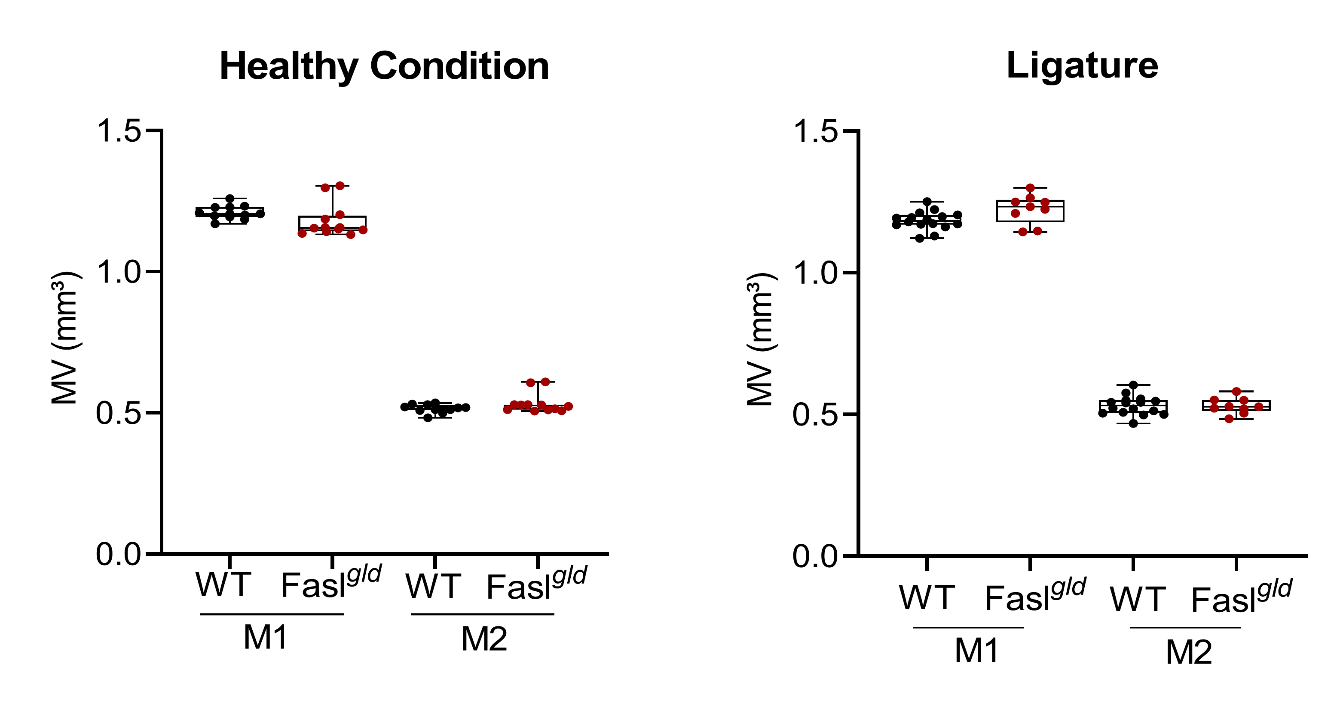


Figure 1S. Under Healthy conditions and upon Ligature induced periodontitis there were no differences regarding the volume of the molars (M1 and M2) for both strains.

**
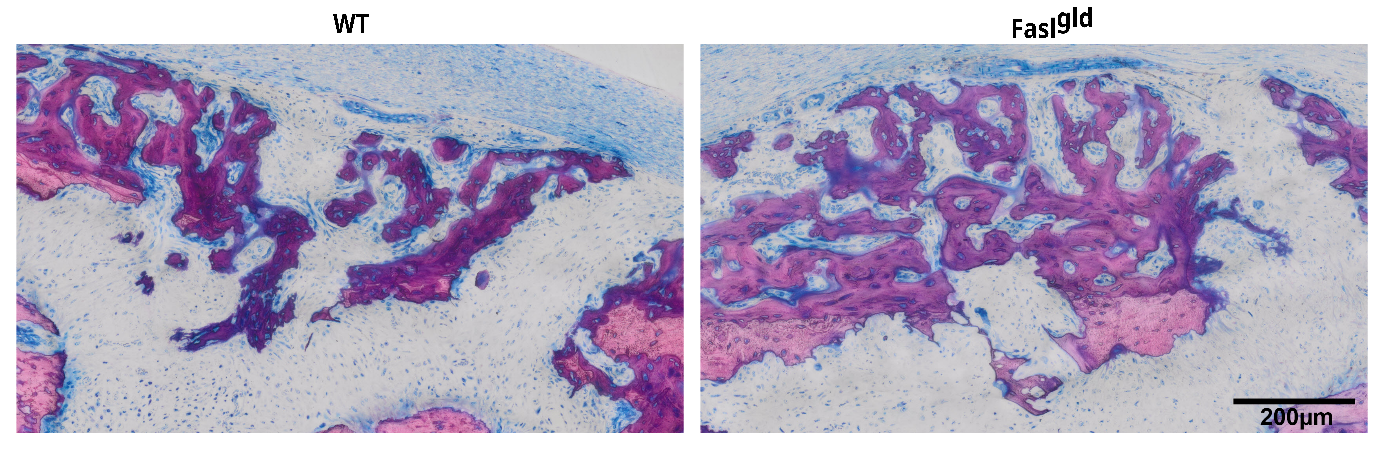
**

Figure 2S. Representative histological sections of the WT and Fasl^gld^ mice displayed fast-growing woven bone originating from the periosteum

**
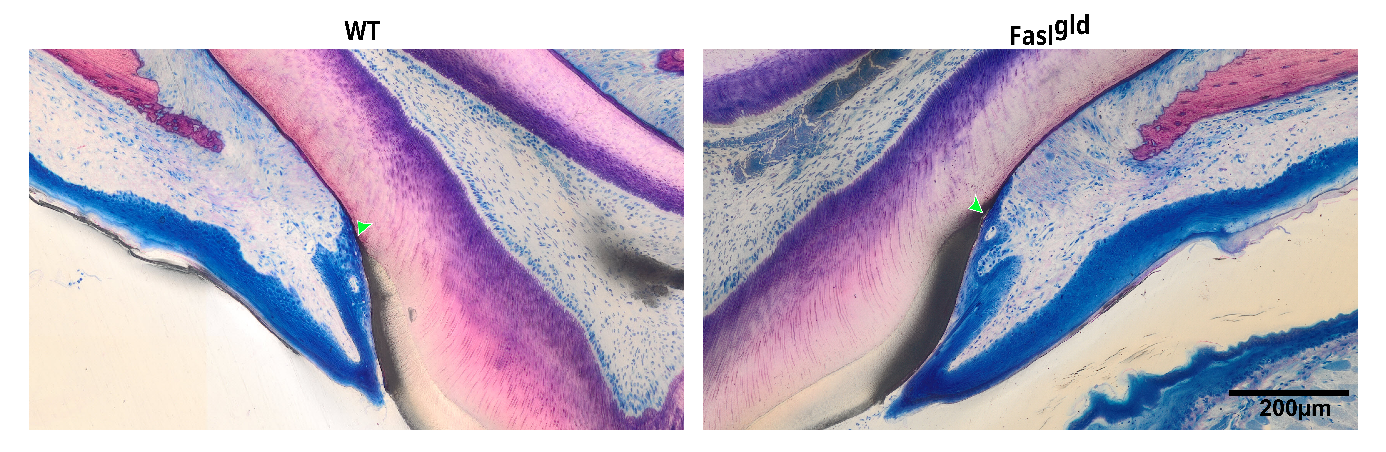
**

Figure 3S. Representative histological sections of the WT and Fasl^gld^ mice displayed the junctional epithelium. Arrowheads point towards the CEJ. The picture represents the mesial part of the first molar.
